# Molecular Mechanisms and Pathophysiology of Ischemia-Reperfusion Injury

**DOI:** 10.3390/ijms19124093

**Published:** 2018-12-18

**Authors:** Arnau Panisello-Roselló, Joan Roselló-Catafau

**Affiliations:** Ischemia-Reperfusion Unit, Experimental Pathology Department, Institut d’Insvestigacions Biomèdiques de Barcelona (IIBB), Spanish Research Council, 08036 Barcelona, Catalonia, Spain; arnau.panisello.rosello@gmail.com

Ischemia-reperfusion injury (IRI) is a major cause of graft loss and dysfunction in clinical transplantation and organ resection. The predominant focus on immunological rejection in this context has rather diverted attention away from IRI and its consequences, despite its association with a huge number of clinical and health issues.

In IRI it is important to distinguish between the two phases of the syndrome, which are differentiated but inseparable. “Ischemia” is the process by which the blood flow is restricted or interrupted for a certain period, and “reperfusion” is the subsequent process by which the blood flow is restored and oxygen enters the organ [1,2]. Total IRI damage is the sum of ischemic insult plus reperfusion insult; these two phenomena are different, and thus provoke different organ/cell responses.

The pathophysiology of IRI is complex and multi-factorial [3]. The mechanisms that underlie it should be studied in depth, based on experimental trials and clinical therapeutic strategies designed to address its repercussions or to prevent its damage [4]. It is important to study IRI from different points of view and at different levels in order to shed light on its complexity and to obtain a holistic understanding of the process.

In the special issue “Molecular Mechanisms and Pathophysiology of Ischemia-Reperfusion Injury”, we report new investigations and strategies for different organs such as liver, brain, heart, and kidney. We also report on new trends and studies that provide an in-depth view of the pathophysiological mechanisms. All in all, this special issue gives a broad overview, ranging from the generalist new trends to the specific avant-garde studies of IRI.

In liver, most reports of IRI are associated with clinical procedures. Several contributions have shown that during the phase of ischemia in cold preservation, cellular mechanisms such as the m-TOR or the ubiquitin proteasome system are already involved [5]. These factors are determinant for the regulation of cytoprotective autophagy and the prevention of apoptosis [6]; the regulation of the inflammation (and the mediators that help to prevent it, such as the vasodilator nitric oxide, NO) is also evidenced in cold preservation, especially when it is associated with the preservation of the glycocalyx, the thin layer of sugars that covers the liver endothelia. The use of a non-toxic compound such as polyethylene glycol 35 as the trigger seems to be beneficial for liver [7].

Still in relation to cold preservation, other markers normally studied in other contexts seem to be gaining relevance. An example is the mitochondrial enzyme ALDH2, which has mostly been studied in ethanol detoxification. ALDH2 activation has been associated with hepatoprotection [8], but in other cases it seems that the process of enzyme inactivation is associated with hepatoprotection; indeed, recent investigations of the blockade of PPARγ [9] or the glutamate receptor mGluR5, which can halt the excitotoxic events, have shown benefits in ischemia alone and in both warm and cold IRI [10].

Some authors propose the use of certain drugs such as thymoquinone to improve IRI, applying a multifactorial approach [11], while others propose to study the specific protection of the bioenergetics of the mitochondria, by adding berberine [12].

Finally, to improve the condition of the liver, some authors propose using new methodological strategies—either hypothermic preconditioning prior to warm IR, which reduces both damage and oxidative indicators [13], or the introduction of new techniques such as dynamic preservation with machine perfusion [14]. This seems a good alternative to static preservation, especially in subnormothermic conditions [15], when the benefits of enzymatic action remain higher than in hypothermic conditions. Currently, this is a hot point in liver preservation using machine perfusion. Finally, we should note that steatosis makes the liver vulnerable to IRI, especially in transplantation and increases the rate of primary failure. In this regard, the activation of the complement in liver I/R may be a critical target in donor livers with mild to moderate steatosis which are being considered for use in transplantation [16].

In contrast to the liver, the research into the pathophysiology of IRI in the brain centers mainly on disease or on surgical procedures other than transplantation. Again, the pathophysiology of IRI involves autophagy and mediators such as the Akt–mTOR pathway, which seems to be a double-edged sword; it may be modulated by the protein kinase cPKCγ, as a therapeutic target [17]. Other authors focus more on the techniques used to counter the brain damage done in IR. In some cases, ancient Chinese medicinal techniques such as acupuncture have palliative effects after a stroke [18].

In other cases, post-ischemic palliative benefits have also been obtained by using more recently-developed drugs such as sevoflurane [19]. The in vivo evaluation of cerebral hemodynamic and oxygen imaging techniques is particularly interesting, due to its cross-linking properties and its potential for use not only in brain but in other organs as well [20].

The modulation of cellular mechanisms in order to prevent or diminish the deleterious effects of IRI has also been studied in kidney, using non-toxic compounds such as polyethylene glycols of different molecular weight, which are present in commercial preservation solutions [21]. Once again, the aim is to alleviate the damage done during ischemia, which will have repercussions for reperfusion. In other cases, drugs are used that mix the broad fields of immunology and IRI in an attempt to reduce the damage [22]. Interestingly, IRI may promote acute kidney injury (AKI) in which epithelial cell cycle involvement has also been suggested [23].

Moving on to the heart, stroke has established itself as a major issue, due to the changes in lifestyles recorded in recent decades. Studies have suggested that old traditions such as the Mediterranean diet may hold some keys to a successful defense against cardiac IRI [24]. Multi-organ studies also show that the human body is a group of communicating vessels and not a set of isolated, unrelated organs [25,26].

Finally, future strategies in IRI prevention should aim to modulate the damage during each of the two phases. First, during ischemia, strategies should focus on the prevention of energy breakdown due to oxygen deprivation and the activation of protective “cell signaling” pathways that occur as the organ’s “self-response” to counter the lack of oxygen [6]; then, during reperfusion, the aim should be the prevention of oxidation processes, in which the mitochondria plays a key role in extending the oxidative damage [27]. These proposals, and the role of other potential factors associated with co-stimulatory pathways and T regulatory cells [28], seem to constitute new challenges for modulating the complex pathophysiology of IRI and organ function [29] in the future.

This special issue covers a wide range of perspectives related to ischemia-reperfusion injury, from the latest tendencies to the most novel therapeutic strategies, some of them using traditional techniques and others using recently developed drugs. Each study provides its own perspective on the issue: some by focusing on ischemia alone, others by studying either warm or cold ischemia, others still in relation to sickness or surgical procedures, and so on. The beauty is that each of these contributions, even though they seem heterogeneous, are part of the big picture presented in this issue of the complex pathophysiology of ischemia-reperfusion injury and the therapeutic perspectives for preventing its deleterious effects.
